# Phytochemical screening and anti-obesity, anti- diabetic and anti-oxidant properties of *Scoparia dulcis* leaf decoction (crude)

**DOI:** 10.6026/97320630019238

**Published:** 2023-03-31

**Authors:** Arulandhu Clara Mary, Christopher Ireen, Periyasamy Vijayalakshmi, Sabapathy Indhu, Sekar Divyabharathi, Kulandaisamy Suregka Felix, Viyagulasamy Daisyrani, Manikkam Rajalakshmi

**Affiliations:** 1 Department of Biotechnology and Bioinformatics, Holy Cross College (Autonomous), Tiruchirappalli, Tamil Nadu, India; 2 DBT-BIF Centre, Holy Cross College (Autonomous), Tiruchirappalli, Tamil Nadu, India; 3 Department of Zoology, Holy Cross College (Autonomous), Tiruchirappalli, Tamil Nadu, India; 4 Department of Business Administration, Holy Cross College (Autonomous), Tiruchirappalli, Tamil Nadu, India; 5 Department of Social Work, Holy Cross College (Autonomous), Tiruchirappalli, Tamil Nadu, India

**Keywords:** Diabetes, *Scoparia dulcis*, phytocompounds, molecular docking

## Abstract

Due to their rising prevalence, diabetes and obesity were both classified as epidemics by the World Health Organization.The natural leaf essence of *Scoparia dulcis* (*S. dulcis*), is used as herbal remedy for diabetes and obesity worldwide. However, the objective of the current research was to examine the effects of consuming commercially available *S. dulcis* porridge on both diabetes and obesity. The S. dulcis plants were collected and the essence was prepared in a traditional way. Phytochemical screening was carried out to identify the secondary metabolites present in the essence. The GC-MS analysis was carried out to identify the bioactive compounds present in the extracts. In order to understand the molecular interaction of identified compounds with selected target proteins from anti-diabetic, anti-oxidant, anti-obesity molecular docking studies was carried out. Results of this docking studies confirmed that identified compounds showed the strong interaction with all the selected target protein. Further experimental analysis is needed to confirm this activity.

## Background:

Diabetes mellitus is one of the most common long-term diseases in metabolic disorders. Diabetes affects 171 million people globally, according to the World Health Organization (WHO). By the year 2025, the total number of individuals with diabetes is predicted to be around 300 million, with this number expected to quadruple by 2030. [1]. Obesity is the leading cause of insulin resistance, which emerges early in the disease's progression and is compensated mostly by hyperinsulinaemia. Children who are overweight, tall, and have a large waist circumference are more likely to have insulin resistance [2]. Type 2 diabetes is brought on by obesity and an insulin deficit [3]. According to the STEPS survey conducted by the World Health Organization in 2002, obesity and Type 2 diabetes were both present in 54.8% of people. The same poll from 2013 showed a prevalence of Type 2 diabetes of 45.8%. There could be 552 million Type 2 diabetics worldwide in 2030, according to estimates [4]. Plants have always been a great source of pharmaceuticals, and they have yielded many important medicines in the past, either directly or indirectly. For example, the traditional strategy of utilizing Galega officinalis resulted in the discovery of Metformin, a commonly used hypoglycemic medication.[5]. Herbal medicines are gaining popularity due to a number of benefits, including a lower incidence of adverse effects at recommended dosages, improved patient tolerance, and acceptability due to a long history of use. In the case of diabetes, the more essential reason is that herbal medicine gives a rational approach of treating the disease as well as many other ailments that are difficult to treat and incurable in more western medical systems [6]. *Scoparia dulcis* (*S. dulcis*) often known as sweet broom weed or Mithipatti (Family: Scrophulariaceae), is a perennial herb that grows in both trophic and subtrophic zones. Grazed grasslands, moist wastelands, and cultivated regions, in particular, have plentiful growth. The plant's leaves are serrated, and the flowers are white. It's used to treat a variety of diseases, and it tastes like sugar, it's been used as a sugar alternative for diabetic patients. *S. dulcis* has been utilized as an anti-diabetic plant in several traditional medicinal systems [6]. In the present study, bioactive compounds present in the *S. dulcis* was identified and their interaction with selected target proteins from Anti-obesity, anti- diabetic and anti-oxidant was identified through molecular docking studies.

## Materials and Methods:

## Collection of plant:

The whole plant of *S. dulcis* was collected from Palur, Trichy District, Tamil Nadu, India in December 2021. The plant was recognized and verified by the Department of Herbarium at St. Joseph's College in Trichy, Tamil Nadu.

## Preparation of Plant Extraction:

The fresh and healthy leaves of S.dulcis were removed carefully from the collected plants and washed with clean water. To avoid the impact of the water remained back after washed. The extra water was allowed to drain off. Then the leaves were ground gently using mortar and pestle. The smooth paste was formed then tied in a muslin cloth and it was kept in a clean bottle to collect the natural essence. The collected essence was stored at 4oC properly for further use.

## Preliminary Phytochemical Screening:

Preliminary phytochemical Screening of the plants was carried out as per the standard methods.

## Test for Phenols (ferric chloride test):

Take 1 millilitre of the essence, 3 millilitres of distilled water, and a few drops of the 5% FeCl3 solution. The existence of phenolic compounds was established by their dark green colour.

## Test for Terpenoids (by salkowski test):

To 1ml of essence of S.dulcis, 2ml of chloroform was added, followed by 3ml of concentrated H2SO4 to form a layer. A reddish brown coloration of the border indicated the presence of terpenoids.

## Test for Tannins:

To 1ml of essence, few drops of 1% FeCl3 solution were added. The blue, black, green or blue green precipitate indicates the presence of tannins.

## Test for Saponins:

1 ml of essence was combined with 2 ml of distilled water and briskly shaken. The existence of saponins was confirmed by prolonged, stable foam.

## Test for Steroids:

In order to create a stable, long-lasting froth, 2ml of distilled water was added to 1ml of plant essence and forcefully shaken. Three drops of olive oil were added to the mixture, which was then briskly shaken. The presence of steroids is shown by the emulsion's formation.

## Test for Flavonoids:

A few drops of H2SO4 were added to 1 millilitre of S.dulcis extract and 1 millilitre of NaOH. Flavonoids were present as indicated by the development of a yellowish brown tint.

## Test for Glycosides:

One drop of a FeCl3 solution was added to one millilitre of glacial acetic acid, one millilitre of the sample's plant essence was mixed thoroughly, and one millilitre of concentrated H2SO4 was then added. The presence of cardiac glycosides was indicated by a brown ring at the interphase.

## Molicsh's Reagent Test:

To 1ml of essence, 5ml of Molisch's reagent and concentrated H2SO4 were added. The violet color indicated presence of glycosides.

## Test for Volatile oils:

To 1ml of essence added 1ml of 90% ethanol, followed by a few drops of FeCl3. The green color formation indicate the presence of volatile oils.

## Test for Carbohydrates:

1ml of concentrated H2SO4 and a few drops of Molisch's reagent were added to 1ml of essence. It was then diluted with 3ml of distilled water after standing for 2 minutes. The presence of carbohydrates is indicated by the red or dim violet colour at the intersection of two layers.

## Test for Reducing Sugar:

Two millilitres of fehling's A and fehling's B solutions were added to two millilitres of essence sample and cooked for one minute. The test samples were cooked in a bath of boiling water for 5-10 minutes. The presence of brick-red precipitate indicates that reducing sugar is present.

## Test for Protein:

 To 1ml of plant essence in the sample, an equivalent volume of biurette reagent was added and the mixture was cooked in a boiling water bath for 2 minutes. The presence of proteins is indicated by the presence of a bluish-green hue.

## Test for polysaccharide:

The essence (0.5g) was dissolved in distilled water and filtered. The filtrate was mixed with iodine solution. The formation of dark blue color indicates the presence of polysaccharides.

## Test for Alkaloids:

A few drops of acetic acid and 1 millilitre of Wagner's reagent were added to 1 millilitre of sample essence. Reddish brown colour production suggests the presence of alkaloids.

## GC-MS) analysis:

Sample preparation:

The natural essence was powdered through air drying. The powder was then mixed with methanol and homogenized. It was then filtered using filter paper and collects the clear sample for GC-MS analysis. All GC-MS measurement were obtained on a ShimadzuQP2020 gas chromatography-mass spectrometer with the following settings: column: GC-MSQP2020 (30m/0.25mm/0.25mum); oven temperature; 50°c, hold for 3 min, ramp to 200oc at 20°C/min, hold for 10 min; injection temperature; 250°c; splitting ratio; 10.0; MS ion source temperature; 200 c; interface temperature: 250°C: total run time: 40.33 min.

## Molecular docking:

## Preparation of receptors:

The 3D X-ray crystallographic structures of the target proteins, Autolysin (PDB ID; 1GVM-A), Protein kinase B/AKT (PDB ID; 1GZN-A), Interleukin 6 (PDB ID; 1IL6-A), Insulin receptor substrate 1 (1IRS-A), Phosphoenol pyruvatecarboxy kinase (PDB ID; 1KHB-A), Tumor necrosis factor (PDB ID; 1NCF-A), Superoxide dismutase (PDB ID; 1SPD-A), Glutathione peraoxidase (PDB ID; 2HE3-A), Insulin receptor (PDB ID; 6CEB-A), Catalase (PDB ID; 1QQW-A) were obtained from Protein Data Bank (PDB). The Discovery Studio Visualizer 2017 R2 Client programme was used to prepare the receptors by eliminating the hetero-atoms and water molecules and adding polar hydrogen atoms.

## Preparation of ligands:

Using ACD/Chemsketch, the structures of *Scoparia dulcis* compounds were sketched and imported in mol2 format. The SDF-formatted 3D structures of 1) 1, 2-Benzenedicarboxylic acid, bis (-methylpropy) ester, 2) Hexadecanoic acid, methyl ester, 3) 2-hexadecen and 4) Methyl stearate was obtained from the PubChem database. All ligands were converted to PDBQT format and stored for use with the PyRx-Virtual screening programme.

## Molecular Docking:

The receptor proteins and ligands were docked using PyRx Version 0.8, which permits preparation of the target protein's binding and screening of the chemical library. Discovery Studio 2017 R2 Client software was used to visualize the results.

## Results and Discussion:

The *S. dulcis* plant leaf decoction was subjected to various tests and analysis in order to study the pharmacological properties of leave decoction against diabetes, obesity and anti-oxidant challenges.

## Phytochemical analysis:

The phytochemical analysis of the crude extract of *S. dulcis* leaf decoction was summarized in the Table 1.

## GC-MS:

The crude decoction prepared from the plant leaves were subjected to GC-MS study and the identified compounds were drawn using Chemsketch.

## Molecular Docking:

The molecular interaction docking studies was performed for the non-sugar compounds found in the plant crude decoction after being found by GC-MS. The interaction between the target protein and the ligands performed with the PyRx version (0.8) with autolysin, AKT, Interleukin, IRS1, PEPCK, TNF-alpha, Superoxide dismutase, glutathione peroxide, Insulin Receptor and Catalase. The binding affinity the RMSD values were summarized in the Table 2. The interaction between the amino acids is indicated in pink and green, for hydrogen bonding and Vander Walls Force with side (S) and main Chain (M) interactions. The Docking results proved that the ligands have very good molecular interactions with the targeted proteins. Autolysin is one of the major enzymes that play a vital role in the virulence of gram-positive organisms. The down regulation of the enzyme can either kill the organism or can neutralize the virulent nature. The serine/ threonine Tyrosine Kinase (AKT) protein is one of the many proteins involved in cell growth and survival and also includes regulation of glucose and lipid metabolism and is expressed in insulin responsive tissues (Huang et al., 2018). By stimulating hexokinase, AKT converts glucose to Glucose- 6 - Phosphate and also regulates glycolysis to produce cellular energy. It also promotes glycogen production by inhibition [7]. Interleukin 6 (IL-6) is a pro inflammatory cytokine, the presence of IL-6 is normal in tissues with normal conditions whereas, in abnormal conditions it leads to inflammation due to irregular production and over exposure, in-case of Type 2 Diabetes Millets (T2DM) it leads to resistance towards insulin. The expression of SOC-3 which is a potent insulin signaling inhibitor is induced by the impaired phosphorylation of insulin Receptor (IR) and Insulin Receptor Substrate (IRS) [8]. Similarly, TNF- alpha is another proinflammatory cytokine which plays a vital role in the development of insulin resistance in T2DM through Serine phosphorylation. The inhibitory treatment strategies of TNF- alpha towards insulin resistance T2DM are being developed [9]. Superoxide Dismutase (SOD) is an Anti-oxidant enzyme that plays a vital role in various Reactive Oxygen Species (ROS) in which the over expression can cause to toxicity. The cellular levels of ROS can be regulated accordingly with antioxidant enzymes and small molecule anti-oxidants. The SOD isozymes (SOD1, SOD2, SOD3) are often liked with the incidence of obesity and diabetes [10]. Glutathione peroxide provides protection against oxidative challenges; inhibit inflammation and oxidant-induced apoptosis in the cellular level. It reduces intracellular hydrogen peroxide and lipid peroxides. Gene knockout mice for Glutathione peroxide in diabetes is been currently developed [11]. Catalase is an essential antioxidant enzyme that controls oxidative stress. It regulates oxidative stress by eliminating cellular hydrogen peroxide to generate water and oxygen. Diabetes mellitus, hypertension, anaemia, vitiligo, Alzheimer's disease, Parkinson's disease, bipolar disorder, cancer, and schizophrenia, among others, are caused by the down regulation of catalase [12]. Thus, each target proteins studies in the above study plays a vital role the regulation of diabetes, obesity and oxidative challenges that occur in the body and can be mitigated with the help of non- sugar moieties available in plants like *Scoparia dulcis*.

## Conclusion:

In the present study, the non-sugar moieties obtained from the *Scoparia dulcis* plant shows efficient bonding with the target proteins which in-turn infers as the antidiabetic, anti-obesity and anti-oxidant properties of the compounds. This can be further subjected to future analysis of the compounds in the path of new sugar supplement production.

## Figures and Tables

**Table 1:** The table shows that the leaf extract of S.dulcis indicated the presence of flavonoids, terpenoids, Phenolic compound, Steroids ,Glycosides ,Saponins, Alkaloids, Molish’test, Volatile oils, Carbohydrate, Reducing sugar and proteins.

**S.No.**	**Test for**	**Result**
1	Tannins	-
2	Flavonoids	+
3	Terpenoids	+
4	Phenolic Compound	+
5	Steroids	+
6	Glycosides	+
7	Saponins	+
8	Alkaloids	+
9	Molish Test	+
10	Volatile oils	+
11	Carbohydrates	+
12	Reducing sugars	+
13	Proteins	+
14	Polysaccharides	-

**Table 2:** Molecular Docking interaction between selected target proteins with selected compounds

**S.no**	**Compound name**	**Binding energy**
	Autolysin	
1	1,2-Benzenedicarboxylic acid.	-5.7
2	2-HEXADECEN-1-OL, 3,7,11,15-TETRAMETHYL-, [R-[R*,R*-(E)]]-	-5.2
3	Hexadecanoic Acid	-4.3
4	Methyl Sterate	-5
	AKT	
1	1,2-Benzenedicarboxylic acid.	-5.3
2	2-HEXADECEN-1-OL, 3,7,11,15-TETRAMETHYL-, [R-[R*,R*-(E)]]-	-4.8
3	Hexadecanoic Acid.	-4.4
4	Methyl Sterate	-4.2
	Interlukin-6	
1	1,2-Benzenedicarboxylic acid	-5.6
2	2-HEXADECEN-1-OL, 3,7,11,15-TETRAMETHYL-, [R-[R*,R*-(E)]]-	-5.2
3	Hexadecanoic Acid	-4.4
4	Methyl Sterate	-4.2
	IRS-1	
1	1,2-Benzenedicarboxylic acid	-4.6
2	2-HEXADECEN-1-OL, 3,7,11,15-TETRAMETHYL-, [R-[R*,R*-(E)]]-	-4.9
3	Hexadecanoic Acid	-4.5
4	Methyl Sterate	-4.7
	PEPCK	
1	1,2-Benzenedicarboxylic acid	-6.3
2	2-HEXADECEN-1-OL, 3,7,11,15-TETRAMETHYL-, [R-[R*,R*-(E)]]-	-4.7
3	Hexadecanoic Acid	-4.9
4	Methyl Sterate	-4.8
	TNF-Alpha	
1	1,2-Benzenedicarboxylic acid	-5.2
2	2-HEXADECEN-1-OL, 3,7,11,15-TETRAMETHYL-, [R-[R*,R*-(E)]]-	-4.3
3	Hexadecanoic Acid	-3.6
4	Methyl Sterate	-3.6
	Superoxide Dismutase	
1	1,2-Benzenedicarboxylic acid	-4.9
2	-HEXADECEN-1-OL, 3,7,11,15-TETRAMETHYL-, [R-[R*,R*-(E)]]-	-5.1
3	Hexadecanoic Acid	-3.6
4	Methyl Sterate	-4.4
	Glutathione Peroxide	
1	1,2-Benzenedicarboxylic acid	-5.2
